# A non-classical synthetic strategy for organic mesocrystals

**DOI:** 10.3389/fchem.2024.1454650

**Published:** 2024-09-16

**Authors:** Shaoyan Wang, Thu Ha Tran, Jia Jia, Yuhua Feng

**Affiliations:** ^1^ CAS Key Laboratory of Materials for Energy Conversion, Shanghai Institute of Ceramics Chinese Academy of Sciences (SICCAS), Shanghai, China; ^2^ Division of Chemistry and Biological Chemistry, School of Physical and Mathematical Sciences, Nanyang Technological University, Singapore, Singapore; ^3^ School of Materials Science and Engineering, Nanyang Technological University, Singapore, Singapore; ^4^ School of Chemistry and Molecular Engineering, Institute of Advanced Synthesis, Nanjing Tech University, Nanjing, China

**Keywords:** organic mesocrystals, solvent exchange, crystal transformation, lattice mismatch, microrod array

## Abstract

Mesocrystals are ordered nanoparticle superstructures, often with internal porosity, which receive much recent research interest in catalysis, energy storage, sensors, and biomedicine area. Understanding the mechanism of synthetic routes is essential for precise control of size and structure that affect the function of mesocrystals. The classical synthetic strategy of mesocrystal was formed via self-assembly of nanoparticles with a faceted inorganic core but a denser (or thicker) shell of organic molecules. However, the potential materials and synthetic handles still need to be explored to meet new applications. In this work, we develop a non-classical synthetic strategy for organic molecules, such as tetrakis (4-hydroxyphenyl) ethylene (TPE-4OH), tetrakis (4-bromophenyl) ethylene (TPE-4Br), and benzopinacole, to produce mesocrystals with composed of microrod arrays via co-solvent-induced crystal transformation. The aligned nanorods are grown epitaxially onto organic microplates, directed by small lattice mismatch between plates and rods. Thus, the present work offers general synthetic handle for establishing well-organized organic mesocrystals.

## 1 Introduction

A mesocrystal is different from a normal crystal in that it is not completely solid and continuous. It is often composed of many aligned nanocrystals with gaps and pores among them. It is similar to crystal in terms of the long-range order over micrometer or even macroscopic distances (Sturm and Cölfen, 2016; Jehannin et al., 2019; Ni et al., 2022). As such, the high surface area of mesocrystals finds wide applications in batteries (Ye et al., 2011; Qiu et al., 2021; Jiang et al., 2022; Yang et al., 2023b), catalysis (Fang et al., 2011; Tachikawa and Majima, 2014; Ji et al., 2022; Yang et al., 2023a), surface enhanced Raman scattering (Ye et al., 2021; Lübkemann-Warwas et al., 2023), sensors (Liu et al., 2021; He et al., 2023), and biomedicine (Ma and Cölfen, 2014; Du et al., 2020).

Mesocrystal is also different from the superlattice of nanoparticles (Macfarlane et al., 2011; Macfarlane et al., 2013), where the position of the nanoparticles is ordered but not necessarily their lattice alignment. Hence, the ordering of the constituent nanocrystals in mesocrystal is its defining characteristic, requiring a special explanation. The understanding would provide a basis for future synthetic efforts and functional designs, attracting great interest in the community (Sturm and Cölfen, 2016).

The ordering could arise from the oriented alignment of crystals, often through additional means: 1) The truncated octahedral PbS nanocrystals were assembled into a 2D superlattice, where the 2 nm oleic acid layer is thin enough not to interfere with the packing with shape-based alignment, but thick enough to prevent complete coalescence (Simon et al., 2012). 2) When the positive (110) facets of BaCO_3_ were selectively blocked by negative polyphosphonate copolymer, the BaCO_3_ nanoparticles assembled into the orderly mesocrystals by asymmetric attachment through the neutral (011) and (020) facets (Yu et al., 2005). 3) In the presence of an external magnetic field (Thomas et al., 2008; Li et al., 2010; Kapuscinski et al., 2020), Fe_3_O_4_ nanocrystals could be directly aligned into mesocrystals based on their magnetic anisotropy (Kapuscinski et al., 2020). 4) The periodic “hole zone” (the non-helical aera) of collagen fiber could be used to adsorb apatite nanocrystals by electrostatic attraction. The c-axis of apatite crystal is almost parallel to the long axis of collagen fiber, so that a mesocrystalline structure would arise from the orderly pores (Nudelman et al., 2010). Similarly, the protein β-sheets from lysozyme were exploited as the basic building blocks for mesoscale assembly (Tao et al., 2017).

The above mesocrystals are mostly inorganic, sometimes involving protein as the co-assembly building blocks. Molecular mesocrystals are rare, possibly because of the lack of facet control for organic crystals, let alone assembly techniques. An exception is the mesh structures that evolved from the C_70_ crystals (Bairi et al., 2016). Which is clearly not a result of assembly.

Recently, we found that the co-solvent exchange in a C_60_ solvate crystal could transform plates into mesh networks (Lei et al., 2019; Wang et al., 2020). The loss of co-solvent destabilizes the original plate lattice, so that the subsequent on-site recrystallization leads to epitaxial growth of rods, eventually giving orderly mesh networks. The product is essentially mesocrystals. Considering the prevalence of co-solvents in molecular crystals, we believe that the co-solvent-induced crystal transformation could serve as a new synthetic strategy and a general platform for constructing organic mesocrystals.

**FIGURE 1:**
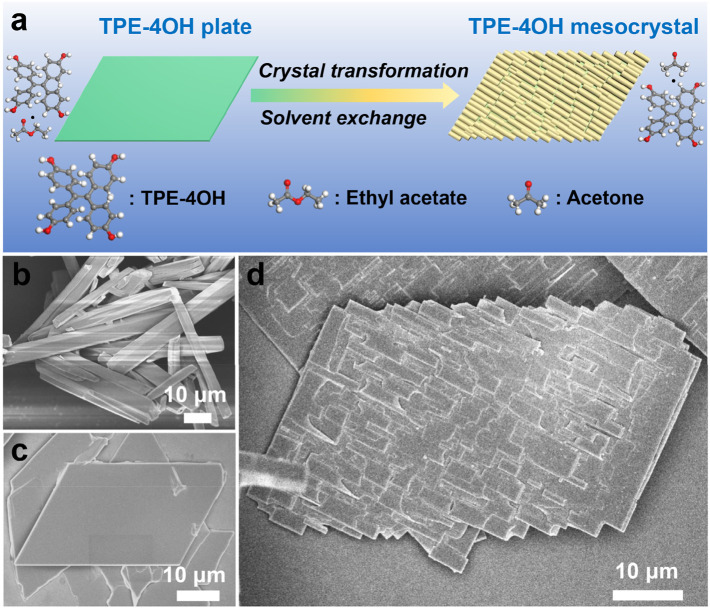
**(A)** Schematics illustrating the transformation of TPE-4OH microplate to microrod arrays. SEM image of **(B)** TPE-4OH microrods and **(C)** TPE-4OH microplates. **(D)** TPE-4OH microrod arrays.

Here, we explore several solvate crystals for preparing organic mesocrystals. Different from the assembly mechanism of traditional mesocrystal, the strategy of co-solvent induced crystal dissolution and reconstruction was adopted. The solvents are put in organic crystals as tunable space-filler and then they are removed to induce strain. By tuning the solvent exchange, the organic crystals undergo crystal transformation. The dissolution of plate and reformation of rods all build upon and rely on the initial form of the organic plates. Moreover, the emerging microrods epitaxially grow on the original plates, resulting a well-arranged mesocrystalline structure (Figure 1A). Not only tetrakis (4-hydroxyphenyl) ethylene (TPE-4OH), but also tetrakis (4-bromophenyl) ethylene (TPE-4Br) and benzopinacole molecules can be used the spontaneous strategy to construct mesocrystals. Hence, the synthetic handle of molecular/organic mesocrystallization provides a new horizon for the generation of porous and well-organized crystalline structures in a much broader and potentially useful way.

## 2 Experimental

### 2.1 Materials

Tetrakis (4-hydroxyphenyl) ethylene (TPE-4OH, 97.0%), Tetrakis (4-bromophenyl) ethylene (TPE-4Br, 97%), and benzopinacole, were purchased from Tokyo Chemical Industry (TCI). Cyclohexane (99%), ethyl acetate (99.5%), *p*-xylene (99%), acetone (99.5%), toluene (99.5%), dioxane (99%), N,N-Dimethylformamide (DMF, 99.8%) were purchased from Sigma-Aldrich. Isopropanol alcohol (IPA, HPLC) was purchased from J.T.Baker^®^ brand. All the chemicals are used without further treatment.

### 2.2 Methods

#### 2.2.1 Synthesis of TPE-4OH microplates and microrods

**FIGURE 2:**
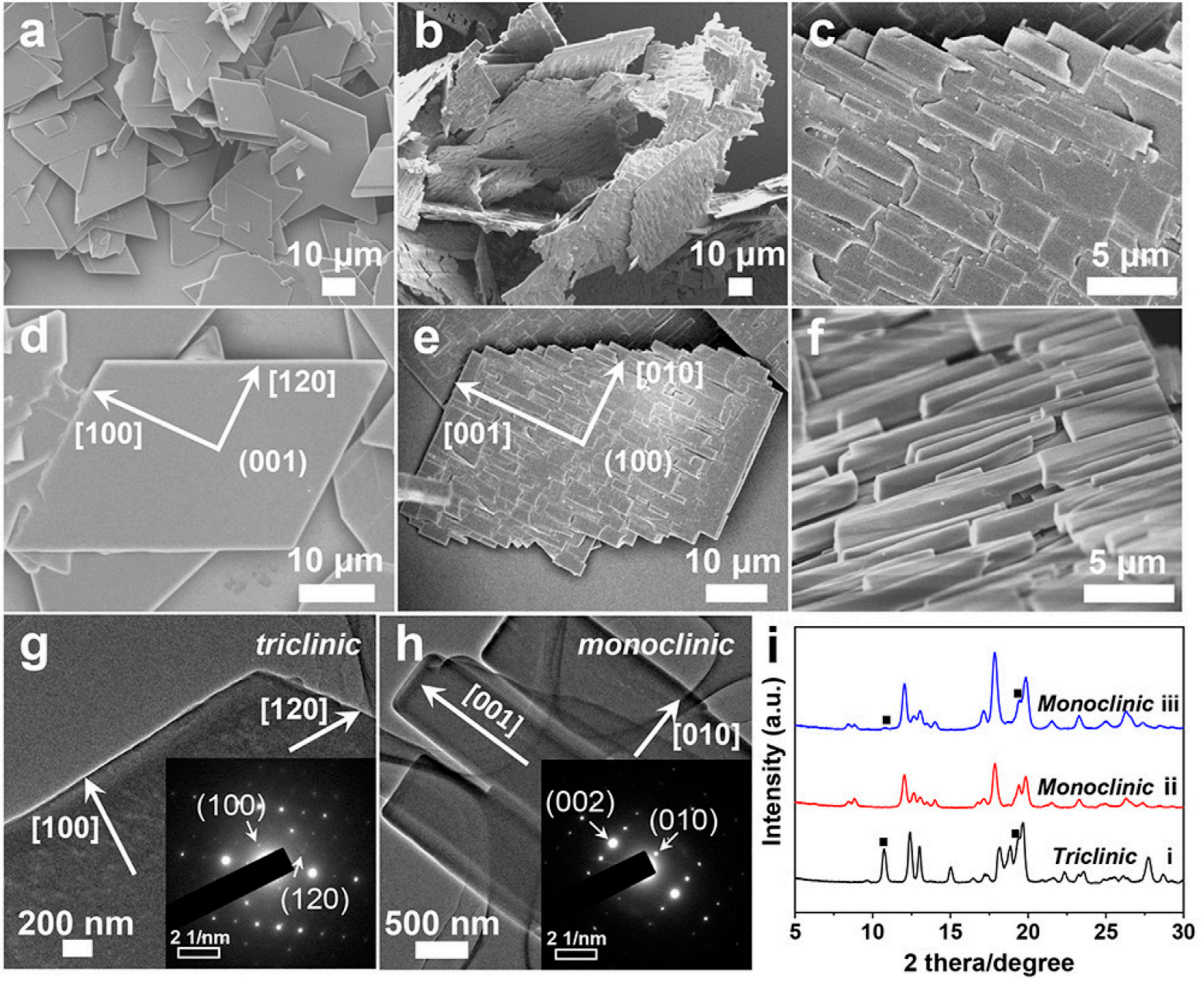
**(A)** SEM images of TPE-4OH microplates. **(B)** SEM images of TPE-4OH mesocrystals at lower concentration of acetone/cyclohexane (v/v = 1:9). **(C)** SEM image of TPE-4OH microrods. **(D,E)** SEM images of single TPE-4OH microplate and mesocrystal. **(F)** The SEM image of mesocrystal at higher concentration of acetone/cyclohexane (v/v = 2:8). **(G)** TEM image of TPE-4OH microplate, the insert shows the SAED patterns of the microplate. **(H)** TEM image of TPE-4OH microrods of mesocrystal, the insert is the SAED patterns of the microrod. **(I)** XRD of TPE-4OH crystals (I) TPE-4OH microplates, (II) TPE-4OH microrods, and (III) TPE-4OH mesocrystals.

The TPE-4OH crystals were prepared by using LLIP method. The cyclohexane was selected as the poor solvent to precipitate TPE-4OH crystals. In a typical synthesis, 12.5 mg TPE-4OH powders were dissolved in 1 mL acetone by sonication for 5 min. Then, 0.5 mL cyclohexane was slowly added into 0.5 mL TPE-4OH/acetone stock solution and kept for 5 min. Then the TPE-4OH microrods were formed at the interface between the cyclohexane and the acetone (Supplementary Figure S1A). In a typical synthesis, 18.7 mg TPE-4OH powders were dissolved in the 1 mL ethyl acetate by sonication for 1 min. Then, 0.5 mL cyclohexane was slowly added into 0.5 mL TPE-4OH/ethyl acetate stock solution and kept for 10 min. Finally, the TPE-4OH microplates were formed at the interface between the cyclohexane and the ethyl acetate (Figure 2A; Supplementary Figure S1B). The Fourier Transform Infrared Spectrometer (FTIR) was also carried out to investigate the incorporated co-solvents (Supplementary Figure S2). The stability of TPE-4OH microplates have shown in Supplementary Figure S3.

The molar ratio of the incorporated solvent for TPE-4OH crystals was determined by thermogravimetric analysis (TGA) performed in a nitrogen atmosphere. It can be expected that two stages of solvent loss should be included in the TPE-4OH solvate crystals, namely, the removal of the solvent molecules at lower temperatures and the subsequent sublimation of the TPE-4OH matrix at higher temperatures. We first performed the TGA analysis for a pure TPE-4OH sample, as shown in Supplementary Figure S4A. It is obvious that the raw TPE-4OH powder starts to lose weight at a temperature of 207°C, which the temperature acts as a reference point. It can be determined that the TPE-4OH microrods contained 21.5% acetone (w/w Supplementary Figure S4B), whereas the TPE-4OH microplates contained 22.1% ethyl acetate (w/w Supplementary Figure S4C). The TGA data of TPE-4OH mesocrystals showed two stages of solvent loss, about 5.3% and 9.9% w/w, respectively (Supplementary Figure S4D).

#### 2.2.2 Synthesis of TPE-4OH mesocrystals

The TPE-4OH microplates were isolated by centrifugation and redispersed in cyclohexane. Then microplates were isolated and immersed in an acetone/cyclohexane mixture (V/V = 0.5/9.5, 1/9, 2/8, 3/7) for several minutes (Supplementary Figure S5). The TPE-4OH mesocrystals were formed after crystal transformation.

#### 2.2.3 Synthesis of TPE-4Br microplates and microwires

**FIGURE 3:**
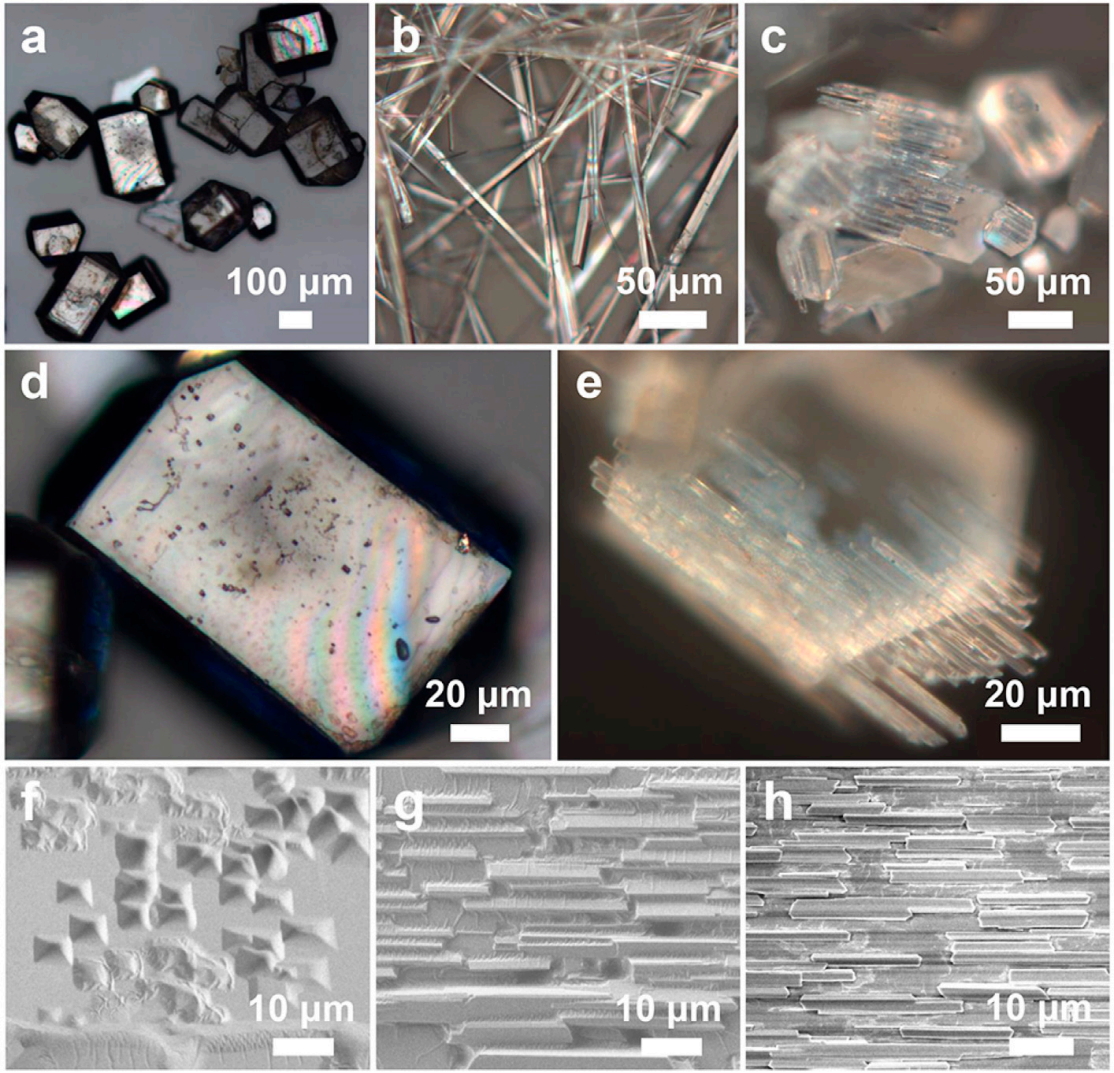
Optical images of **(A,D)** TPE-4Br microplates, **(B)** TPE-4Br microwires, **(C,E)** TPE-4Br mesocrystals. SEM images of the crystal transformation from microplate to microrod arrays. **(F)** *t* = 1 min; **(G)** 3 min; **(H)** 10 min.

The TPE-4Br crystals were prepared by using the LLIP method. In a typical synthesis of *p*-xylene-rich microplates, 50 mg TPE-4Br powders were dissolved in 3 mL *p*-xylene by sonication. 0.5 mL IPA (poor solvent) was slowly injected into the stock solution of TPE-4Br in *p*-xylene (16.7 mg/mL, 0.5 mL), which formed an interface between the stock solution and the IPA. Then, the vial was kept undisturbed for 5 min. Next, the two solvents were mixed together by sonication for 30 s. Afterward, the mixture was stored in an incubator at 25°C for 12 h. Finally, the colorless microplates were formed at the bottom of the vial (as shown in Figures 3A,D; Supplementary Figure S6A). In the synthesis of toluene-rich microwires, 1.0 mL IPA was slowly injected into a stock solution of TPE-4Br in toluene (30 mg/mL, 0.5 mL). The same procedure as above. Then the microrods were formed at the bottom of the vial (Figure 3B; Supplementary Figure S6B).

#### 2.2.4 Synthesis of TPE-4Br mesocrystals

The TPE-4Br microplates were formed in *p*-xylene/IPA mixture as mentioned above. TPE-4Br microplates were isolated from the mother liquor, then the TPE-4Br microplates were incubated in an IPA-toluene mixture (V/V = 2:1), and the mixtures were allowed to stand for 10 min (Figure 3C; Supplementary Figure S6C).

The raw TPE-4Br powder starts to lose weight at a temperature of 235°C (Supplementary Figure S7A). It shows that the TPE-4Br microplates contained 13.7% *p*-xylene (w/w Supplementary Figure S7B), and the TPE-4Br microwires contained 2.1% toluene (w/w Supplementary Figure S7C). The TGA data of TPE-4Br mesocrystals showed 1.7% solvent loss, which was assigned to the toluene (Supplementary Figure S7D). The stability of TPE-4Br microplates have shown in Supplementary Figures S8, S9. TPE-4Br microplates contained 12.1% *p*-xylene (w/w Supplementary Figure S10) after one round of purification by IPA solution.

#### 2.2.5 Synthesis of benzopinacole microplates and microrods

20 mg benzopinacole powders were dissolved in 1.5 mL dioxane solution by sonication for 5 min. Then, 2 mL IPA was slowly added into 1 mL benzopinacole, resulting in rhomboid microplates in IPA-dioxane interfaces after 5 min (Supplementary Figures S11A,B). In the procedure of synthesis of benzopinacole microrods, 20 mg benzopinacole powders were dissolved in 1 mL DMF solution. Then, 2 mL IPA was slowly added into 1 mL benzopinacole, resulting in microrods after 5 min (Supplementary Figure S11C).

#### 2.2.6 Synthesis of benzopinacole mesocrystals

The benzopinacole microplates were formed in dioxane/IPA mixture. The benzopinacole microplates were isolated by centrifugation, then the benzopinacole microplates were incubated in IPA-DMF mixture (V/V = 3:1), and the mixtures were allowed to stand for 10 min (Supplementary Figure S11D).

### 2.3 Characterization

The morphologies and sizes of the samples were examined using field-emission scanning electron microscopy (FESEM, JEOL 7600F) at an acceleration voltage of 5 kV. Prior to analysis, the samples were coated with a thin gold layer using an Edwards Sputter Coater. TEM images were obtained using a JEOL JEM-2100 electron microscope at an accelerating voltage of 200 kV. One drop of the as-prepared colloidal dispersion was deposited on a carbon-coated copper grid, and dried under high vacuum. The X-ray diffraction (XRD) patterns were measured by Bruker D8 Advance Powder with Cu Ka radiation (λ = 1.5406 Å). We operated in the 2θ range from 5° to 40°, by using the samples spin-coated on the surface of a quartz substrate. The single crystal XRD parameter of TPE-4Br/toluene nanorod was obtained by Bruker Kappa CCD Diffractometer. The single crystal images with growth directions were obtained by Bruker Smart APEXII SC-XRD. Thermogravimetric analysis (TGA) curve was obtained using a TGA Q500 thermogravimetry analyzer. The optical images were obtained Olympus BX51 optical microscope. The Raman studies were measured under ambient conditions by using 488 nm excitation with an integration time of 10 s. IR spectra were taken on an FTIR Perkin Elmer Frontier. For the UV-vis absorption spectra, the sample was dropped on a quartz wafer and tested by UV-vis-NIR Lambda 950.

## 3 Results and Discussion

In this work, the TPE-4OH is selected as a model system (Figure 1), as it is known to give solvate crystals (Tanaka et al., 2000). Firstly, the TPE-4OH solvate crystals were prepared by using the liquid-liquid interfacial precipitation (LLIP) method (Shrestha et al., 2013). Microrods formed at the interface between cyclohexane and a solution of TPE-4OH in acetone, whereas uniform 2D rhomboid microplates were obtained in the ethyl acetate/cyclohexane system (Figures 1C, 2A,D). Both crystals have smooth surfaces, and are stable in the preparative solution for a prolonged period. They were isolated by centrifugation and no purification was carried out to reduce the loss of embedded solvent.

As characterized by scanning electron microscopy (SEM), the average length and diameter of the TPE-4OH rods were 43.7 μm and 4.6 μm, respectively (Figure 1B; Supplementary Figures S1A,D). Thermogravimetric analysis (TGA) showed that the microrods contained 21.5% solvent (w/w, Supplementary Figure S4B). The average length and width of the TPE-4OH microplates were about 30.0 μm and 16.8 μm (Supplementary Figures S1B,E). They contained about 22.1% solvent (w/w, Supplementary Figure S4C). On the bases of the literature (Tanaka et al., 2000), the co-solvents embedded in these two types of crystals are expected to be acetone and ethyl acetate, respectively. The molar ratios of the TPE-4OH: solvent (Calculated by TGA) in rods and plates are 1.9:1 and 1.2:1 (Table 1), respectively, which match the results in the literature (Tanaka et al., 2000) (Supplementary Figure S2).

**TABLE 1:** Dependence of crystal morphologies on the embedded solvents.

TPE-4OH crystal	Ethyl acetate% (*w/w*)	Acetone% (*w/w*)	n_ethyl acetate_/n_acetone_	n_ethyl acetate_/n_TPE-4OH_	n_acetone_/n_TPE-4OH_
Microplates	22.1	0	—	1.9:1	—
Microrods	0	21.5	—	—	1.2:1
Mesocrystals	5.3	9.9	0.35:1	0.28:1	0.8:1

The FTIR was also carried out to investigate the incorporated co-solvents. The IR spectrum of the TPE-4OH microplates showed one peaks at around 1,375 cm^−1^, which can be ascribed to symmetrical C-H bending vibration of ethyl acetate (Supplementary Figure S2) (Nolin and Jones, 1956). The IR spectra of the TPE-4OH microrods and TPE-4OH mesocrystals presented two peaks at 2,921 and 2,953 cm^−1^, indicating the symmetrical and asymmetrical C-H stretch vibration of the acetone (Dellepiane and Overend, 1966; Hudson et al., 2018), respectively (Supplementary Figure S2). The ethyl acetate molecule and acetone interact with TPE-4OH by hydrogen bonds (Tanaka et al., 2000), while the ethyl acetate molecules parallel to *cb* of TPE-4OH, and the acetone molecules may be included in channels along the *a*-axis or lie between the layers of TPE-4OH (Tanaka et al., 2000; Toda, 2002).

The fact that the TPE-4OH crystals are intact in their respective mother liquor suggests that there is an equilibrium between the embedded solvent molecules and those in the solution. In other words, they have the same driving force (Wang et al., 2020). As the solvent ratios change during the purification and drying processes, it is expected that the driving force will change accordingly. When the TPE-4OH microplates were exposed to air, many small holes generated on the surface (Supplementary Figure S3A). A similar situation occurs when the plates are immersed in a solution, that is, miscible with its internal solvent, for example, the microplates are purified with cyclohexane solution. It showed that the microplates were weathering with random pores (Supplementary Figure S3B). It agrees with the expectation that the entropy increase should be larger when the solvent molecules are mixed with cyclohexane than with air molecules, which are much less in number. Thus, the desolvation rate in solution will be very fast. Once the embedded solvent is desorbed, there is no chance to go back.

The formation of pores should be unfavorable because it exposes unfavorable facets, and increases the surface area and thus, the surface energy. However, if we can provide an embedded driving force (incorporating other solvent molecules) in the desolvation process, the morphologies of organic crystals will not be deformed or damaged, but will be effectively regulated. When the microplates were dispersed in an acetone/cyclohexane (V/V = 0.5/9.5) solution, no crystal transformation took place (Supplementary Figure S5A). It indicates that the driving force of the embedded solvent is too low, resulting in the solvent loss being less serious. However, the embedded driving force is still insufficient to transform the crystal morphology.

To have a more controlled solvent exchange process for preparing organic mesocrystals, the solvated TPE-4OH microplates were dispersed in a solution with a precise concentration of acetone (10%) in cyclohexane. This particular concentration is selected by experiments, so that the loss of co-solvent becomes moderate, which provides a platform for continuous transformation. Finally, the organic mesocrystals with the microrod array morphologies were obtained in the transformative solution (acetone/cyclohexane). The microrods laid on the microplates and abreast arranged in the same direction to form the microrod arrays, which are perpendicular to the short edges of the rhomboid microplate and extend to the outside of long edges to appear zigzag morphology (Figures 1D, 2B,C,E).

Detailed temporal evolution showed that at *t* = 2 min, short microrods (5.3 μm and 1.5 μm) appeared on the microplate surface (Figures 2C,E; Supplementary Figures S1C,F). The microrods only occur in one direction, all vertical to the short edges of the microplate, suggesting epitaxial growth. Notably, the short edges of the plates remain intact and no transformation occurs. At 8 min, the rods extended in length and width (5.5 μm and 1.8 μm), and parts of short edges transformed to microrod arrays. It appears that the rectangular holes were mainly determined by the interlacing of the rods (Supplementary Figure S5B), not by the position of the random pores with circular cross-sections formed during desolvation.

For the transformation of the TPE-4OH plate to the mesocrystals composed of microrod arrays, we think that it should be a recrystallization process in acetone-cyclohexane mixed solvent system. Due to the miscibility of acetone, cyclohexane and ethyl acetate, when the crystal of TPE-4OH plate was dispersed in the acetone-cyclohexane mixed solvent, the incorporated ethyl acetate in TPE-4OH would be dissolved into the solution. At the same time, the TPE-4OH around the vacancy formed by the escape of ethyl acetate would also be dissolved into the solution due to its solubility in acetone. The continuous dissolution would cause the saturation of TPE-4OH in acetone-cyclohexane co-solvent, driving the occurrence of the heterogeneous nucleation and epitaxial growth of TPE-4OH on the surface of the TPE-4OH plate. The formation of the microrod should be resulted by the incorporation of acetone solvent during the growth process, which is the normal phenomena in organic crystals.

On the above basis, the possible molecular reaction process should involve three steps: 1) The dissolution of the incorporated ethyl acetate in the acetone-cyclohexane mixed solvent; 2) The dissolution of the TPE-4OH molecules around the dissolved ethyl acetate; 3) The uniform multi-site nucleation of TPE-4OH in saturated solution on the surface of TPE-4OH plate; 4) The epitaxial growth of TPE-4OH-acetone solvated microrods. The repeat of step 1-4 would finally cause the formation of TPE-4OH mesocrystal composed of microrod arrays.

Control experiments showed that this recrystallization process can only occur at acetone/cyclohexane ratio higher than 0.1. When the ratio of acetone to cyclohexane was increased to 2:8, the transformation was much faster. The higher solubility of TPE-4OH microplates in the acetone/cyclohexane mixture led to the rapid recrystallization, giving larger rods (10.7 and 1.9 μm) on the mesocrystals (Figure 2F; Supplementary Figure S5C). The holes became larger and the short edges of the microplates had been completely consumed, leaving the array architecture, but the underlying microplate can still be recognized. The TPE-4OH mesocrystals included two solvent peaks (5.4% and 9.9% by TGA, Supplementary Figure S4D), which are close to the peaks of ethyl acetate and acetone in the neat solvents, respectively. The combined ratio of the co-solvents is lower than the ratios achieved with the respective neat solvents. The relative molar ratio of ethyl acetate: TPE-4OH decreases from the 1.9:1 in the microplates, to 0.28:1 in the mesocrystals (Table 1). Hence, some of the ethyl acetate is lost as acetone is included during the crystal transformation.

Further increasing the acetone/cyclohexane ratio to 3:7, the TPE-4OH microplates were broken with random fat microrods (11.4 μm and 2.8 μm) in the solution. Actually, some of the scattered fragments are still composed of microrod arrays with the same direction (Supplementary Figure S5D). It indicates that the microplates are cracked, likely due to the strains arising from the transformation.

When dispersed the organic crystals in another solvent with low concentration, the driving force is insufficient to extract the internal solvent of the crystals. When the driving force is too large, the rapid desolvation would normally lead to weathering or crumbling of the lattice. Only with the right solvent ratio and procedure, were the driving force of the removal and inclusion of solvent molecules regulated and hence, the mesocrystals were obtained under moderate solvent exchange. However, the solvent exchange does not necessarily generate the mesocrystals, but it can be obtained only in the case of lattice dependence. Thus, the TPE-4OH crystals were characterized by XRD to explore the dependence of morphology.

The X-ray diffraction (XRD) patterns confirmed that the TPE-4OH microplates have *triclinic* phase (Figure 2I-I). The TPE-4OH microrods were indexed to the *monoclinic* phase as shown in Figure 2I-II (Tanaka et al., 2000). After transformation at 2:8 solvent ratio, the XRD patterns of the mesocrystals resembled the *monoclinic* structure of the TPE-4OH microrods. The remaining weak peaks (marked by black square) are assigned as the residual TPE-4OH microplates (Figure 2I-III).

The selected area electron diffraction (SAED) pattern of a typical TPE-4OH microplate and microrod (inset in Figures 2G,H) further confirms the *triclinic* phase and *monoclinic* phase, respectively. Measured from SAED patterns, the lattice distance *d*_*010*_ of the rod is 10.19 Å. Whereas the *d*_*001*_ of the *monoclinic* rods on the mesocrystals is 9.94 Å. The *d*_*100*_ of the *triclinic* plates is 9.12 Å, and *d*_*120*_ of the plates is 5.54 Å. The lattice mismatching rate between [001] direction of the rod and the [100] direction of the plate is 8.2%. The lattice matching gives rise to a low strain along the growth direction of the rod, which promotes nucleation. On the lateral direction, the high lattice mismatching rate (45.6%) between the [010] direction of the rod and the [120] direction of the plate would generate high strain, preventing conformal coating of one on the other, resulting in the island growth. We believe that once the rod sticks to the plate, the (001) facet of the *triclinic* plate matches the (100) facet of the *monoclinic* rod in the [001] direction. As the original plate gradually dissolves, the microrod arrays eventually lead to mesocrystals.

We note that the growth of a rod on a plate would differ from the coating of one crystal on another, in particular regarding to the interface. When a 2D lattice sits on another, mismatch at any direction would cause strain. However, when a rod sits on a 2D lattice, the initial line of contact would be important for nucleation. Once stuck to each other, lattice matching will lead to crystal epitaxial growth. However, it is difficult to align in one dimension, especially for assembly, because of its limiting factors such as identification, driving force and angle. However, mesocrytsls can be achieved in the epitaxial growth system, which is also the source of mesomorphic order. The TPE-4OH mesocrystal still keeps its crystalline nature. It indicates that the TPE-4OH mesocrystal is a large ordered crystal composed of many small crystals, which conforms to the definition of a mesocrystal (Jehannin et al., 2019).

In essence, the crystal transformation is akin to ripening, except that one type of crystal is dissolved to form a closely related, but different crystal. In contrast, the dissolved and re-crystallized materials in ripening have the same composition. Moreover, the onsite recrystallization differs from the typical ripening. It is the critical factor in fitting the newly generated rods in/on the initial framework of rhomboid plates, rather than forming new particles in the solution.

It is really important that this strategy for preparing organic mesocrystals was not a one-off and a fluke, but a universal synthetic handle. Morphological transformation can occur not only on small crystals, i.e., TPE-4OH crystals, but also on large TPE-4Br and benzopinacole crystals. The colorless TPE-4Br microplates (Figures 3A,D; Supplementary Figure S6A) were formed in IPA-*p*-xylene interface by the LLIP method (Shrestha et al., 2013). The average length and width of the microplates are 190 μm and 156 μm, respectively (Supplementary Figure S6D). When the *p*-xylene was substituted by toluene, 1D TPE-4Br microwires with average diameters of 6 μm and lengths of 144 μm were obtained with smooth surfaces (Figure 3B; Supplementary Figures S6B,E).

To achieve the TPE-4Br mesocrystals, the TPE-4Br microplates were isolated by centrifugation and redispersed in toluene-IPA (V/V = 1:2) solution. At 10 min, TPE-4Br microplates were transformed into microrod arrays. From the optical and SEM images, the oriented microrod arrays are paralleled to the long side of the plate, giving a uniform size with a length of around 18 μm and a diameter of 1.9 μm (Figures 3C,E; Supplementary Figures S6C,F). It appears that rods are epitaxially grown on the plate. The ordered array structures with overall plate shape indicate the on-site recrystallization, resulting in the organic mesocrystals (Figure 3E).

**FIGURE 4:**
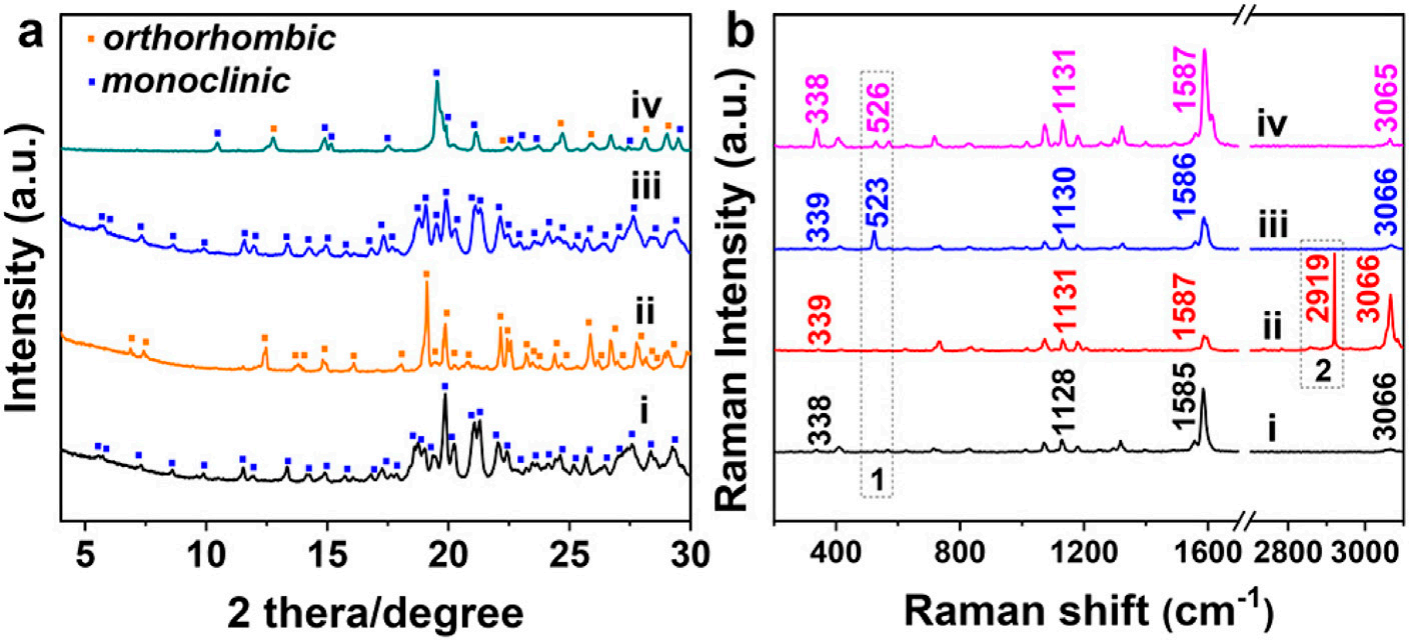
The XRD **(A)** and Raman spectra **(B)** of (I) TPE-4Br powder, (II) TPE-4Br/*p*-xylene microplates, (III) TPE-4Br/toluene microwires, and (IV) TPE-4Br microwire arrays.

The X-ray diffraction (XRD) patterns of TPE-4Br microplates (Figure 4A and ii) were indexed as primitive *orthorhombic* structures, matching that of the TPE-4Br solvate crystals reported in the literature (Tanaka et al., 2000). The TPE-4Br mesocrystals showed similar XRD patterns to the TPE-4Br rods, which have *monoclinic* system (parameters ca. *a* = 20.38 Å, *b* = 9.72 Å, *c* = 23.85 Å, *β* = 92.09°). However, the remaining weak peaks of TPE-4Br mesocrystals were assigned as the residual plates (Figure 4A and iv). The XRD data was consistent with the mesocrystal morphology containing lots of microrods with an overall plate shape. It is similar to the classic mesocrystal, which still retains the crystal characteristics after the formation of an organic mesocrystal (Sturm and Cölfen, 2016). From the TGA data, the TPE-4Br plates have 13.7% *p*-xylene (w/w, Supplementary Figure S7B). After transformation, the TPE-4Br mesocrystals contain 1.7% toluene with the disappearance of *p*-xylene peak (Supplementary Figure S7D). In comparison to the TPE-4Br microwires (2.1%) synthesized in neat toluene solution (Supplementary Figure S7C), the solvent content in mesocrystals is lower. The results indicate that the environmental change provides a driving force for the solvent exchange, leading to co-solvent-induced crystal transformation, in which one type of crystal is dissolved to form a closely related, but different crystal.

Further exploring the influence of solvent environment changes on crystal morphology, the TPE-4Br crystals remained intact in their respective mother liquor (Supplementary Figures S8A,D). The TPE-4Br microwires were stable after one centrifugation-redispersion cycle in IPA (Supplementary Figure S8E), and even exposed to air and vacuum conditions (Supplementary Figure S8F). On the other hand, the TPE-4Br microplates did not show obvious structural change after prolonged storage in the mother liquor (e.g., several weeks, Supplementary Figure S9). However, the TPE-4Br microplates started to show pores when the plates were exposed to air (Supplementary Figure S8C). When the plates were isolated by centrifugation and redispersed in IPA, many irregularly diamond-like particles appeared on the surface and extended to the outside of the plates, resulting in an uneven surface (Supplementary Figure S8B). TGA data showed that the *p*-xylene contents were declined by 1.6% after one round of purification by IPA solution (Supplementary Figure S10). The transformation cannot occur in the mother liquor, because the high concentration of the *p*-xylene would cause the process of solvent inclusion to have the same driving force as that of the desolvation process. The formations of pores or rough surfaces reflect the degree of structural relaxation to release the internal strain. The rate of loss appears to be less in air or vacuum than in an IPA solution. It agrees with our previous results that the entropy increase should be smaller when the *p*-xylene are exposed to air molecules than to IPA, which are much more in number (Wang et al., 2020). Thus, when the equilibrium is disturbed by the environment, the desolvation process occurs.

The Raman spectra of the TPE-4Br crystals shown that the characteristic peak of *p*-xylene disappeared, but the characteristic peak of toluene appeared during the TPE-4Br mesocrystals formation (Figure 4B). The peaks at 338, 714, 1,128, and 3,066 cm^−1^ can be attributed to the characteristic bands of TPE-4Br molecules (Li et al., 2016; Bai et al., 2017). The TPE-4Br microplates have one peak at 2,919 cm^−1^ (Figure 4B-I), which correspond well with CH_3_ asymmetric stretching vibrational modes of *p*-xylene. For the TPE-4Br microwires (Figure 4B-I), the peak at 523 cm^−1^ should originate from the x-sensitive of toluene (Bood and Carlsson, 1995; Gustafsson, 1996). After crystal transformation, the characteristic peak (526 cm^−1^) of toluene presented in the spectrum of the TPE-4Br mesocrystals, while the *p*-xylene peak (2,919 cm^−1^) vanished (Figure 4B-IV). Thus, the Raman data support solvent exchange during crystal transformation.

To further explore the morphological changes of TPE-4Br microplates in transformative solution (toluene-rich IPA solution), we captured the intermediate during the crystal formation (Figures 3F–H). At 1 min, the plates generated many random holes on the surface of the TPE-4Br plate (Figure 3F). The holes show inverse pyramids with quadrilateral cross-sections. These inverse pyramids are well aligned with the plates, similar to the faceted holes of the C_60_ crystals (Wang et al., 2010; Wang et al., 2020). With the transformation going on, many small rods generated on the surface and covered the faceted holes at 3 min (Figure 3G). These microrods have the same growth direction, that is, parallel to the long side of the plate. At 10 min, the microrods extended in length and their face-to-face connection eventually gave the orderly mesocrystal structures (Figure 3H). The formation process indicated that the microplates were etched in the transformative solution, and then the dissolved TPE-4Br materials on-site recrystallized on the original microplates to form the microrod arrays.

**FIGURE 5:**
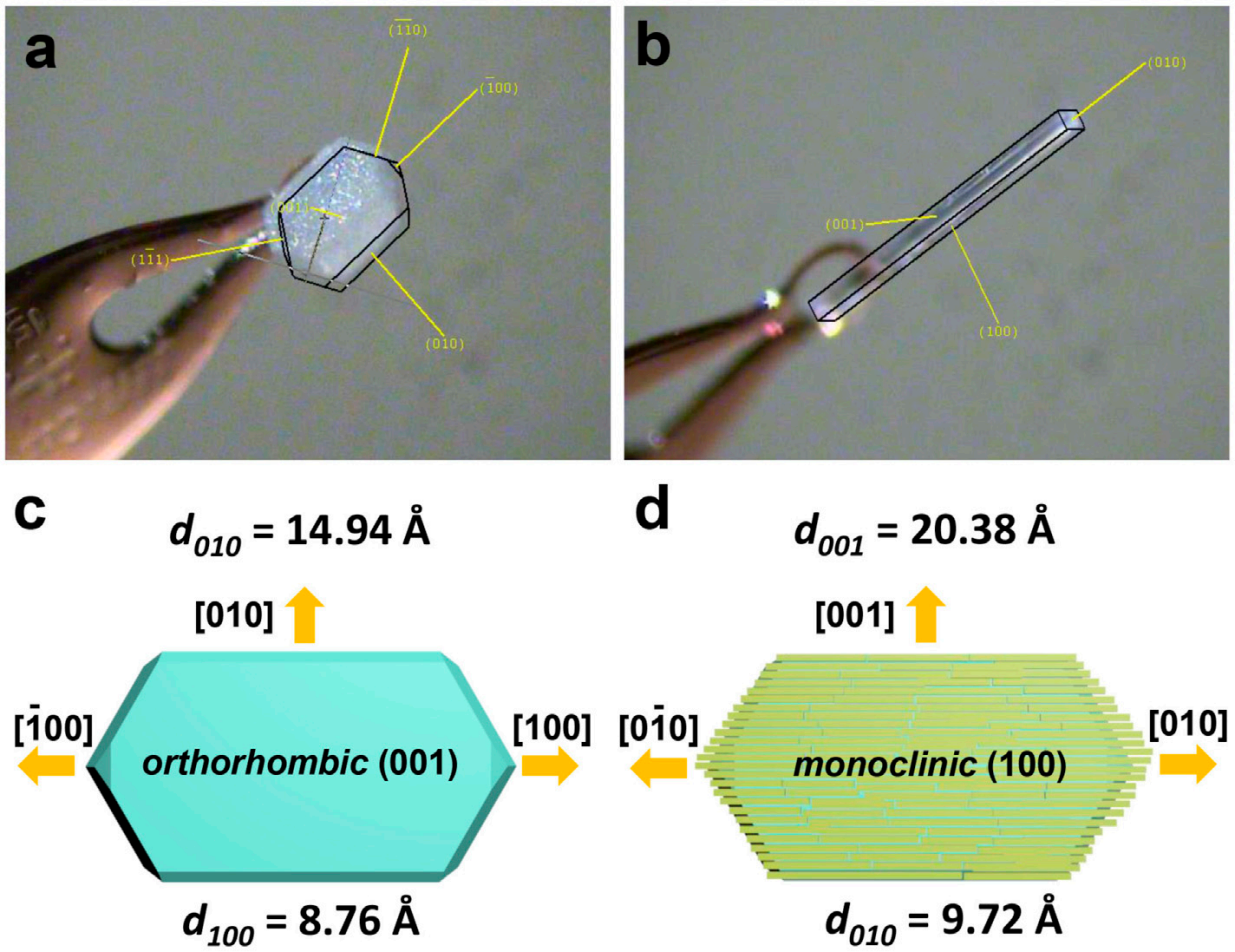
The optical images of **(A)** TPE-4Br microplate and **(B)** TPE-4Br microwire, which were captured during single crystal XRD characterization. **(C,D)** Crystal orientation of *orthorhombic* microplate and *monoclinic* microrod.

In order to investigate the reasons for the orderliness of mesocrystal structure, single crystal XRD of TPE-4Br microplate and TPE-4Br microwire was carried out (Figures 5A,B). The epitaxial relationship between the microplate and the microwire was studied by analyzing the crystal facet and lattice spacing. The TPE-4Br microplate showed a characteristic *orthorhombic* phase; its top surface was bound by (001) planes. The growth direction along the longitude of the microplate is [100] (Figure 5A). The TPE-4Br microwire was confirmed with the *monoclinic* phase, with its long axis pointed at the [010] direction (Figure 5B). Thus, it appears that the [010] direction of the microwire is aligned with [100] of the microplate (Figure 5A). The lattice spacing *d*_*100*_ of the plate was determined from the phase data to be 8.76 Å (Tanaka et al., 2000), whereas the lattice spacing *d*_*010*_ of the microrods was 9.72 Å (Figures 5C,D, CIF file, ESI). Accordingly, the lattice mismatch between the [100] of microplate and the [010] of the rod is 9.8%, resulting in epitaxial growth of the microrod arrays (Figure 5D). Nevertheless, the lattice spacing of the microplate at [010] direction and the microrod at [100] direction is *d*_*010*_ = 14.94 Å and *d*_*010*_ = 20.38 Å, respectively. The large lattice mismatching (26.7%) would generate high strain. Once the rod sticks to the plate, the (001) facet of the *orthorhombic* plate matches the (100) facet of the *monoclinic* rod at the [010] direction and gives rise to island growth at [001] direction, giving rod shape. Thus, the orderliness of mesocrystals stems from epitaxial growth.

Our previous work proved that the slow exchange of co-solvent in C_60_ plates would lead to on-site recrystallization (Wang et al., 2020). The *m*-xylene-rich rods epitaxially grow on the CCl_4_-rich C_60_ plate, resulting in the mesh networks by crystal transformation. The topological change is similar to the formation of organic mesocrystals. The key factor is the diffusion rate of the solvent. If the diffusion rate is fast, the dissolved materials will crystallize into rods in the solution instead of *in-situ* recrystallization relying on the initial crystals (Supplementary Figure S5D). Thus, precise control of the solvent composition and concentration in the solution will inhibit crystal weathering and slow the speed of solvent exchange, resulting in recrystallization in a short distance.

To understand the causes of mesocrystal formation, we identify three core factors of the synthetic strategy: 1) The organic molecules (host molecules) can form the solvates with the solvent molecules (guest molecules); 2) Different solvates give different crystal morphologies; 3) Choosing one solvate crystal as a template, and solvent exchange is carried out to transform it. The newly generated crystal relies on the framework of the original crystal, which is gradually etched for the growth of the new type. The lattice matching degree of the two crystals is high and promotes the formation of orderly mesocrystal.

To further confirm the versatility of the present design strategy, we verified the cases by altering the material to benzopinacole. The rhomboid benzopinacole microplates can be formed at the interface between IPA and a solution of benzopinacole in dioxane and microrods can grow at the IPA-DMF interfaces (Supplementary Figures S10A–C). Then, we transferred benzopinacole microplates to DMF-IPA (V/V = 1:3) solution for 10 min. Many microrods were generated on the surface of microplates and paralleled to one side of plates, resulting in the benzopinacole mesocrystals (Supplementary Figure S10D). The fact that the solvent-induced on-site recrystallization can be applied to different organic crystals, proves the generality of the synthetic handle.

## 4 Conclusion

It is common for solvated organic crystals (Yelgaonkar et al., 2020; Ding et al., 2023) to contain different solvents to form different morphologies, which provides a favorable platform for exploring the generality of organic mesocrystals. We create conditions to place the organic crystals in the co-solvent environment and carry out the solvent exchange slowly in the organic crystals by tuning the composition and concentration of the solvents. In comparison to the irreversible desolvation in gas phase, the results obtained in the solution-phase processes can be more precisely controlled to realize crystal transformation. The principle of mesocrystallization delivers a new mechanism, and the microrod arrays obtained by the solvent-induced on-site recrystallization are a more orderly representation of mesocrystals. In view of the fact that molecular crystals, such as drug molecules, fluorescent molecules, and high-energy molecules, have few synthetic methods for fabricating mesocrystals. Thus, the new synthetic strategy for organic mesoporous or mesocrystals will bring great application prospects.

## Data Availability

The raw data supporting the conclusions of this article will be made available by the authors, without undue reservation.

## References

[B1] Bai Y. Yu Z. Liu R. Li N. Yan S. Yang K. (2017). Pressure-Induced crystallization and phase transformation of *para*-xylene. Sci. Rep. 7, 5321–5410. 10.1038/s41598-017-05639-9 28706305 PMC5509709

[B2] Bairi P. Minami K. Nakanishi W. Hill J. P. Ariga K. Shrestha L. K. (2016). Hierarchically structured fullerene C_70_ cube for sensing volatile aromatic solvent vapors. ACS Nano 10, 6631–6637. 10.1021/acsnano.6b01544 27341124

[B3] Bood J. Carlsson H. (1995). Raman and infrared absorption spectroscopy for tissue diagnostics. Lund Rep. Atomic Phys., 60–65. Available at: https://lup.lub.lu.se/luur/download?func=downloadFile-&recordOId=2260049&fileOId=2297088.

[B4] Dellepiane G. Overend J. (1966). Vibrational spectra and assignment of acetone, ααα acetone-d3 and acetone-d6. Spectrochim. Acta 22, 593–614. 10.1016/0371-1951(66)80091-7

[B5] Ding X. Wei C. Wang L. Yang J. Huang W. Chang Y. (2023). Multicomponent flexible organic crystals. SmartMat 5, e1213. 10.1002/smm2.1213

[B6] Du W. Liu T. Xue F. Cai X. Chen Q. Zheng Y. (2020). Fe_3_O_4_ mesocrystals with distinctive magnetothermal and nanoenzyme activity enabling self-reinforcing synergistic cancer therapy. ACS Appl. Mat. Interfaces 12, 19285–19294. 10.1021/acsami.0c02465 32249558

[B7] Fang J. Ding B. Gleiter H. (2011). Mesocrystals: syntheses in metals and applications. Chem. Soc. Rev. 40, 5347–5360. 10.1039/C1CS15043J 21769374

[B41] Gustafsson M. (1996). Spectroscopic studies of tissue using near-infrared Raman microscopy. Lund Rep. At. Phys. 207, 30–31.

[B8] He Q. Wang B. Liang J. Liu J. Liang B. Li G. (2023). Research on the construction of portable electrochemical sensors for environmental compounds quality monitoring. Mat. Today Adv. 17, 100340. 10.1016/j.mtadv.2022.100340

[B42] Hudson R. L. Gerakines P. A. Ferrante R. F. (2018). IR spectra and properties of solid acetone, an interstellar and cometary molecule. Spectrochim. Acta - A: Mol. Biomol. Spectrosc. 193, 33–39. 29216579 10.1016/j.saa.2017.11.055

[B9] Jehannin M. Rao A. Colfen H. (2019). New horizons of nonclassical crystallization. J. Am. Chem. Soc. 141, 10120–10136. 10.1021/jacs.9b01883 31173682

[B10] Ji Y. Zhou H. Liu S. Kang T. Zhang Y. Chen W. (2022). Isolating single Sn atoms in CuO mesocrystal to form ordered atomic interfaces: an effective strategy for designing highly efficient mesocrystal catalysts. Small 18, 2203658. 10.1002/smll.202203658 36161498

[B11] Jiang Y. Guo S. Li Y. Hu X. (2022). Rapid microwave synthesis of carbon-bridged Nb_2_O_5_ mesocrystals for high-energy and high-power sodium-ion capacitors. J. Mat. Chem. A 10, 11470–11476. 10.1039/D2TA01574A

[B12] Kapuscinski M. Munier P. Segad M. Bergstrom L. (2020). Two-stage assembly of mesocrystal fibers with tunable diameters in weak magnetic fields. Nano Lett. 20, 7359–7366. 10.1021/acs.nanolett.0c02770 32924498 PMC7587140

[B13] Lei Y. Wang S. Lai Z. Yao X. Zhao Y. Zhang H. (2019). Two-dimensional C_60_ nano-meshes via crystal transformation. Nanoscale 11, 8692–8698. 10.1039/C8NR09329F 30742169

[B14] Li F. Ma Z. Fang W. Wang S. Li T. Sun C. (2016). Influence of cation and CH⋯ Br hydrogen bond in benzene–bromobenzene mixture on stimulated Raman scattering. Optik 127, 5347–5350. 10.1016/j.ijleo.2016.03.053

[B15] Li L. Yang Y. Ding J. Xue J. (2010). Synthesis of magnetite nanooctahedra and their magnetic field-induced two-/three-dimensional superstructure. Chem. Mat. 22, 3183–3191. 10.1021/cm100289d

[B16] Liu J. Sun L. Li G. Hu J. He Q. (2021). Ultrasensitive detection of dopamine via electrochemical route on spindle-like α-Fe_2_O_3_ Mesocrystals/rGO modified GCE. Mat. Res. Bull. 133, 111050. 10.1016/j.materresbull.2020.111050

[B17] Lübkemann-Warwas F. Morales I. Bigall N. C. (2023). Recent advances in functional nanoparticle assemblies. Small Struct. 4, 2300062. 10.1002/sstr.202300062

[B18] Ma M.-G. Cölfen H. (2014). Mesocrystals–applications and potential. Curr. Opin. Colloid Interface Sci. 19, 56–65. 10.1016/j.cocis.2014.03.001

[B19] Macfarlane R. J. Jones M. R. Lee B. Auyeung E. Mirkin C. A. (2013). Topotactic interconversion of nanoparticle superlattices. Science 341, 1222–1225. 10.1126/science.1241402 23970559

[B20] Macfarlane R. J. Lee B. Jones M. R. Harris N. Schatz G. C. Mirkin C. A. (2011). Nanoparticle superlattice engineering with DNA. Science 334, 204–208. 10.1126/science.1210493 21998382

[B21] Ni B. Gonzalez-Rubio G. Colfen H. (2022). Self-assembly of colloidal nanocrystals into 3D binary mesocrystals. Acc. Chem. Res. 55, 1599–1608. 10.1021/acs.accounts.2c00074 35679581

[B22] Nolin B. Jones R. N. (1956). The infrared absorption spectra of deuterated esters: ii. ethyl acetate. Can. J. Chem. 34, 1392–1404. 10.1139/v56-178

[B23] Nudelman F. Pieterse K. George A. Bomans P. H. H. Friedrich H. Brylka L. J. (2010). The role of collagen in bone apatite formation in the presence of hydroxyapatite nucleation inhibitors. Nat. Mat. 9, 1004–1009. 10.1038/nmat2875 PMC3084378 20972429

[B24] Qiu X. Wang X. He Y. Liang J. Liang K. Tardy B. L. (2021). Superstructured mesocrystals through multiple inherent molecular interactions for highly reversible sodium ion batteries. Sci. Adv. 7, eabh3482. 10.1126/sciadv.abh3482 34516887 PMC8442931

[B25] Shrestha L. K. Ji Q. Mori T. Miyazawa K. I. Yamauchi Y. Hill J. P. (2013). Fullerene nanoarchitectonics: from zero to higher dimensions. Chem. Asian J. 8, 1662–1679. 10.1002/asia.201300247 23589223

[B26] Simon P. Rosseeva E. Baburin I. A. Liebscher L. Hickey S. G. Cardoso‐Gil R. (2012). PbS–organic mesocrystals: the relationship between nanocrystal orientation and superlattice array. Angew. Chem. Inter. Ed. 51, 10776–10781. 10.1002/anie.201204583 23011885

[B27] Sturm E. V. Cölfen H. (2016). Mesocrystals: structural and morphogenetic aspects. Chem. Soc. Rev. 45, 5821–5833. 10.1039/C6CS00208K 27503260

[B28] Tachikawa T. Majima T. (2014). Metal oxide mesocrystals with tailored structures and properties for energy conversion and storage applications. NPG Asia Mater 6, e100. 10.1038/am.2014.21

[B29] Tanaka K. Fujimoto D. Altreuther A. Oeser T. Irngartinger H. Toda F. (2000). Chiral inclusion crystallization of achiral tetrakis (p-halophenyl) ethylenes with achiral guest compounds. J. Chem. Soc. Perkin Trans. 2, 2115–2120. 10.1039/B003473H

[B30] Tao F. Han Q. Liu K. Yang P. (2017). Tuning crystallization pathways through the mesoscale assembly of biomacromolecular nanocrystals. Angew. Chem. Inter. Ed. 56, 13440–13444. 10.1002/anie.201706843 28841270

[B31] Thomas J. M. Simpson E. T. Kasama T. Dunin-Borkowski R. E. (2008). Electron holography for the study of magnetic nanomaterials. Acc. Chem. Res. 41, 665–674. 10.1021/ar700225v 18459804

[B32] Toda F. (2002). Chiral inclusion crystals constructed with achiral host and achiral guest molecules. Enantiomer 7, 59–65. 10.1080/10242430212198 12108635

[B33] Wang H. Zhang Z. Wong L. M. Wang S. Wei Z. Li G. P. (2010). Shape-controlled fabrication of micro/nanoscale triangle, square, wire-like, and hexagon pits on silicon substrates induced by anisotropic diffusion and silicide sublimation. ACS Nano 4, 2901–2909. 10.1021/nn1000996 20405908

[B34] Wang S. Lai Z. Tran T. H. Han F. Su D. Wang R. (2020). Solvent exchange as a synthetic handle for controlling molecular crystals. Carbon 160, 188–195. 10.1016/j.carbon.2019.11.028

[B35] Yang J. Zhang M. Chen M. Zhou Y. Zhu M. (2023a). Oxygen vacancies in piezoelectric ZnO twin-mesocrystal to improve peroxymonosulfate utilization efficiency via piezo-activation for antibiotic ornidazole removal. Adv. Mat. 35, 2209885. 10.1002/adma.202209885 36644889

[B36] Yang Z. Wang B. Chen Y. Zhou W. Li H. Zhao R. (2023b). Activating sulfur oxidation reaction via six-electron redox mesocrystal NiS_2_ for sulfur-based aqueous batteries. Nat. Sci. Rev. 10, nwac268. 10.1093/nsr/nwac268 PMC10171633 37181097

[B37] Ye J. Liu W. Cai J. Chen S. Zhao X. Zhou H. (2011). Nanoporous anatase TiO_2_ mesocrystals: additive-free synthesis, remarkable crystalline-phase stability, and improved lithium insertion behavior. J. Am. Chem. Soc. 133, 933–940. 10.1021/ja108205q 21142068

[B38] Ye Y. Chen C. Li W. Guo X. Yang H. Guan H. (2021). Highly sensitive W_18_O_49_ mesocrystal Raman scattering substrate with large-area signal uniformity. Anal. Chem. 93, 3138–3145. 10.1021/acs.analchem.0c04516 33523629

[B39] Yelgaonkar S. P. Campillo-Alvarado G. Macgillivray L. R. (2020). Phototriggered guest release from a nonporous organic crystal: remarkable single-crystal-to-single-crystal transformation of a binary cocrystal solvate to a ternary cocrystal. J. Am. Chem. Soc. 142, 20772–20777. 10.1021/jacs.0c09732 33236628

[B40] Yu S.-H. Cölfen H. Tauer K. Antonietti M. (2005). Tectonic arrangement of BaCO_3_ nanocrystals into helices induced by a racemic block copolymer. Nat. Mat. 4, 51–55. 10.1038/nmat1268 15608647

