# Supplementary material for: A non-classical synthetic strategy for organic mesocrystals
Source: Front Chem. 2024 Sep 16;12:1454650. doi: 10.3389/fchem.2024.1454650 (PMC11439792; doi:10.3389/fchem.2024.1454650)
Supplement: Supplementary file 1 [file DataSheet1.docx]

Supplementary Material

**A Non-Classical Synthetic Strategy for Organic Mesocrystals**

Shaoyan Wang^1,3*^, Thu Ha Tran ^3^, Jia Jia^2^, Yuhua Feng^2*^

*^1^CAS Key Laboratory of Materials for Energy Conversion, Shanghai Institute of Ceramics Chinese Academy of Sciences (SICCAS), Shanghai, 200050, China*

*^2^nstitute of Advanced Synthesis, School of Chemistry and Molecular Engineering, Jiangsu National Synergetic Innovation Center for Advanced Materials, Nanjing Tech University, 30 Puzhu South Road, Nanjing 211816, China*

*^3^Division of Chemistry and Biological Chemistry, School of Physical & Mathematical Sciences, Nanyang Technological University, 21 Nanyang Link, 637371, Singapore*

*^4^School of Materials Science and Engineering, 50 Nanyang Avenue, 639798, Nanyang Technological University, Singapore*

*E-mail:* [*wangshaoyan@mail.sic.ac.cn*](mailto:wangshaoyan@mail.sic.ac.cn) *(S. Wang***);* [*ias_yhfeng@njtech.edu.cn*](mailto:ias_yhfeng@njtech.edu.cn) *(Y. Feng***)*

**Material and methods**

**Materials**

Tetrakis(4-hydroxyphenyl)ethylene (TPE-4OH, 97.0%), Tetrakis(4-bromophenyl)ethylene (TPE-4Br, 97%), and benzopinacole, were purchased from Tokyo Chemical Industry (TCI). Cyclohexane (99%), ethyl acetate (99.5%), *p*-xylene (99%), acetone (99.5%), toluene (99.5%), dioxane (99%), N,N-Dimethylformamide (DMF, 99.8%) were purchased from Sigma-Aldrich. Isopropanol alcohol (IPA, HPLC) was purchased from J.T.Baker® brand. All the chemicals are used without further treatment.

**Methods**

**Synthesis of TPE-4OH microplates and microrods.** The TPE-4OH crystals were prepared by using the LLIP method.^1^ In a typical synthesis, 12.5 mg TPE-4OH powders were dissolved in 1 mL acetone by sonication for 5 min. Then, 0.5 mL cyclohexane was slowly added into 0.5 mL TPE-4OH/acetone stock solution and kept for 5 min. Then the TPE-4OH microrods were formed at the interface between the cyclohexane and the acetone (Figure S1a). The cyclohexane was selected as the poor solvent to precipitate TPE-4OH crystals. In a typical synthesis, 18.7 mg TPE-4OH powders were dissolved in the 1 mL ethyl acetate by sonication for 1 min. Then, 0.5 mL cyclohexane was slowly added into 0.5 mL TPE-4OH/ethyl acetate stock solution and kept for 10 min. Finally, the TPE-4OH microplates were formed at the interface between the cyclohexane and the ethyl acetate (Figure 2a, S1b). The Fourier Transform Infrared Spectrometer (FTIR) was also carried out to investigate the incorporated co-solvents (Figure S2). The stability of TPE-4OH microplates have shown in Figure S3.

The molar ratio of the incorporated solvent for TPE-4OH crystals was determined by thermal gravimetric analysis (TGA) performed in a nitrogen atmosphere. It can be expected that two stages of solvent loss should be included in the TPE-4OH solvate crystals, namely, the removal of the solvent molecules at lower temperatures and the subsequent sublimation of the TPE-4OH matrix at higher temperatures. We first performed the TGA analysis for a pure TPE-4OH sample, as shown in Figure S4a. It is obvious that the raw TPE-4OH powder starts to lose weight at a temperature of 207 °C, which the temperature acts as a reference point. It can be determined that the TPE-4OH rods contained 21.5% acetone (w/w Figure S4b), whereas the TPE-4OH microplates contained 22.1% ethyl acetate (w/w Figure S4c). The TGA data of TPE-4OH mesocrystals showed two stages of solvent loss, about 5.3% and 9.9% w/w, respectively (Figure S4d).

**Synthesis of TPE-4OH mesocrystals.** The TPE-4OH microplates were isolated by centrifugation and redispersed in IPA. Then microplates were isolated and immersed in an acetone/cyclohexane mixture (V/V = 0.5/9.5, 1/9, 2/8, 3/7) for several minutes (Figure S5). The TPE-4OH mesocrystals were formed after crystal transformation.

**Synthesis of TPE-4Br microplates and microwires.** The TPE-4Br crystals were prepared by using the LLIP method. In a typical synthesis of *p*-xylene-rich microplates, 50 mg TPE-4Br powders were dissolved in 3 mL *p*-xylene by sonication. 0.5 mL IPA (poor solvent) was slowly injected into the stock solution of TPE-4Br in *p*-xylene (16.7 mg/mL, 0.5 mL), which formed an interface between the stock solution and the IPA. Then, the vial was kept undisturbed for 5 min. Next, the two solvents were mixed together by sonication for 30 s. Afterward, the mixture was stored in an incubator at 25 °C for 12 h. Finally, the colorless microplates were formed at the bottom of the vial (as shown in Figure 3a, d, S6a). In the synthesis of toluene-rich microwires, 1.0 mL IPA was slowly injected into a stock solution of TPE-4Br in toluene (30 mg/mL, 0.5 mL). The same procedure as above. Then the microwires were formed at the bottom of the vial. (Figure 3b, S6b).

**Synthesis of TPE-4Br mesocrystals.** The TPE-4Br plates were formed in *p*-xylene/IPA mixture as mentioned above. TPE-4Br plates were isolated from the mother liquor, then the TPE-4Br plates were incubated in an IPA-toluene mixture (V/V = 2:1), and the mixtures were allowed to stand for 10 min (Figure 3c, S6c).

The raw TPE-4Br powder starts to lose weight at a temperature of 235 °C (Figure S7a). It shows that the TPE-4Br microplates contained 13.7% *p*-xylene (w/w Figure S7b), and the TPE-4Br microwires contained 2.1% toluene (w/w Figure S7c). The TGA data of TPE-4Br mesocrystals showed 1.7% solvent loss, which was assigned to the toluene (Figure S7d). The stability of TPE-4Br microplates have shown in Figure S8, S9. TPE-4Br microplates contained 12.1% *p*-xylene (w/w Figure S10) after one round of purification by IPA solution.

**Synthesis of benzopinacole microplates and microrods.** 20 mg benzopinacole powders were dissolved in 1.5 mL dioxane solution by sonication for 5 min. Then, 2 mL IPA was slowly added into 1 mL benzopinacole, resulting in rhomboid microplates in IPA-dioxane interfaces after 5 min (Figure S11a, b). In the procedure of synthesis of benzopinacole microrods, 20 mg benzopinacole powders were dissolved in 1 mL DMF solution. Then, 2 mL IPA was slowly added into 1 mL benzopinacole, resulting in microrods after 5 min (Figure S11c).

**Synthesis of benzopinacole mesocrystals.** The benzopinacole microplates were formed in dioxane/IPA mixture. The benzopinacole microplates were by centrifugation, then the benzopinacole microplates were incubated in IPA-DMF mixture (V/V = 3:1), and the mixtures were allowed to stand for 10 min (Figure S11d).

**Characterization.**

The morphologies and sizes of the samples were examined using field-emission scanning electron microscopy (FESEM, JEOL 7600F) at an acceleration voltage of 5 kV. Prior to analysis, the samples were coated with a thin gold layer using an Edwards Sputter Coater. TEM images were obtained using a JEOL JEM-2100 electron microscope at an accelerating voltage of 200 kV. One drop of the as-prepared colloidal dispersion was deposited on a carbon-coated copper grid, and dried under high vacuum. The X-ray diffraction (XRD) patterns were measured by Bruker D8 Advance Powder with Cu Ka radiation (λ = 1.5406 Å). We operated in the 2θ range from 5 to 40°, by using the samples spin-coated on the surface of a quartz substrate. The single crystal XRD parameter of TPE-4Br/toluene nanorod was obtained by Bruker Kappa CCD Diffractometer. The single crystal images with growth directions were obtained by Bruker Smart APEXII SC-XRD. Thermogravimetric analysis (TGA) curve was obtained using a TGA Q500 thermogravimetry analyzer. The optical images were obtained Olympus BX51 optical microscope. The Raman studies were measured under ambient conditions by using 488 nm excitation with an integration time of 10 s. IR spectra were taken on an FTIR Perkin Elmer Frontier. For the UV-vis absorption spectra, the sample was dropped on a quartz wafer and tested by UV-vis-NIR Lambda 950.


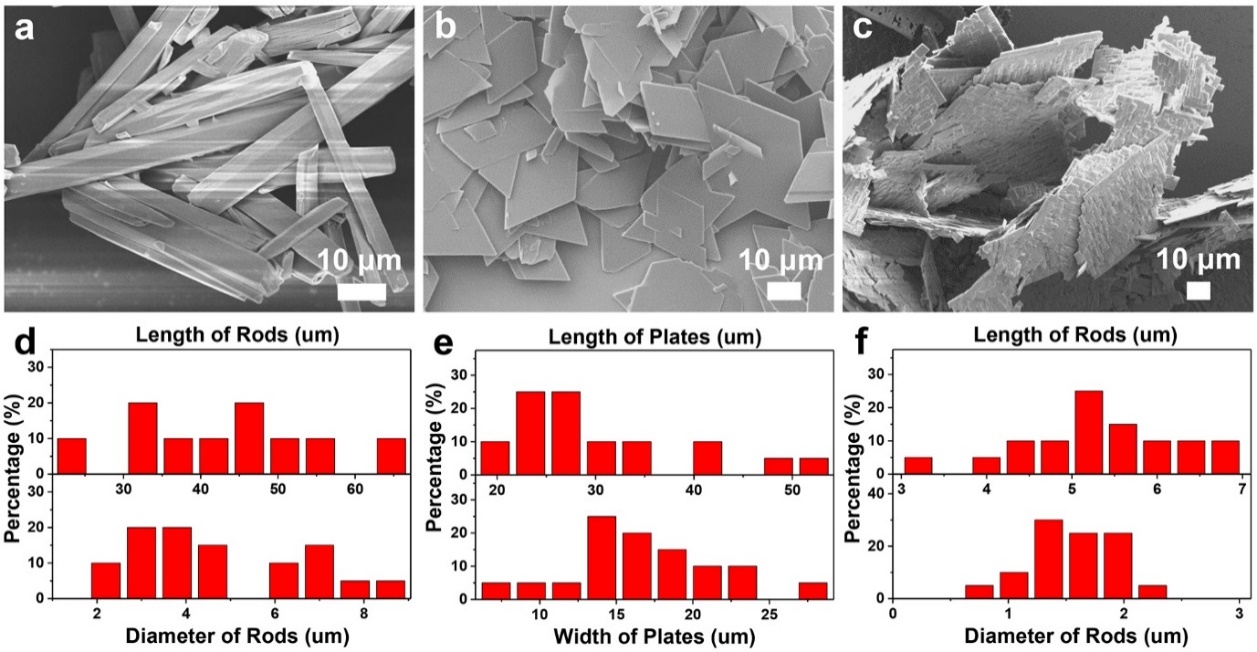


**Figure S1.** The SEM images of TPE-4OH crystals. (a) The SEM image of TPE-4OH microrods, (b) TPE-4OH microplates, and TPE-4OH mesocrystals. (d-f) The size distribution of TPE-4OH microrods, microplates, and microrods of mesocrystals, respectively.


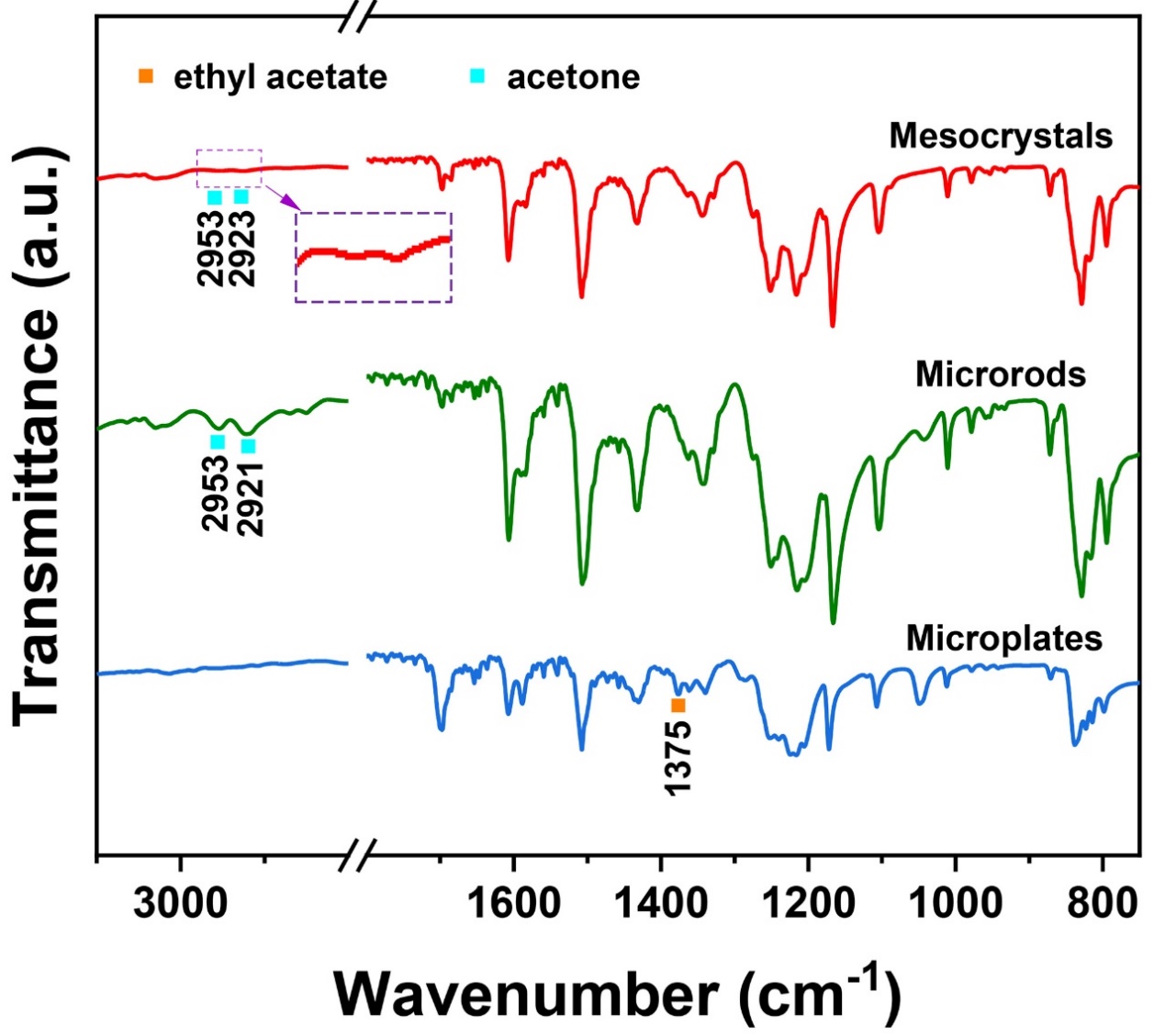


**Figure S2.** The FTIR spectra of TPE-4OH microplates, TPE-4OH microrods, and TPE-4OH mesocrystals.


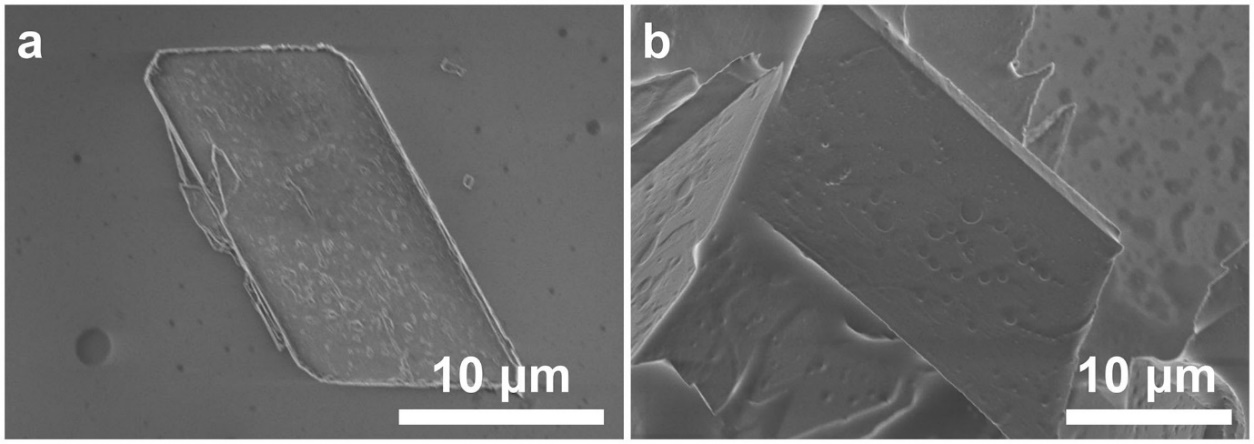


**Figure S3.** The SEM images of TPE-4OH microplates. a) The TPE-4OH microplates were exposed to air for 2 h. b) The TPE-4OH microplates were purified with cyclohexane solution.


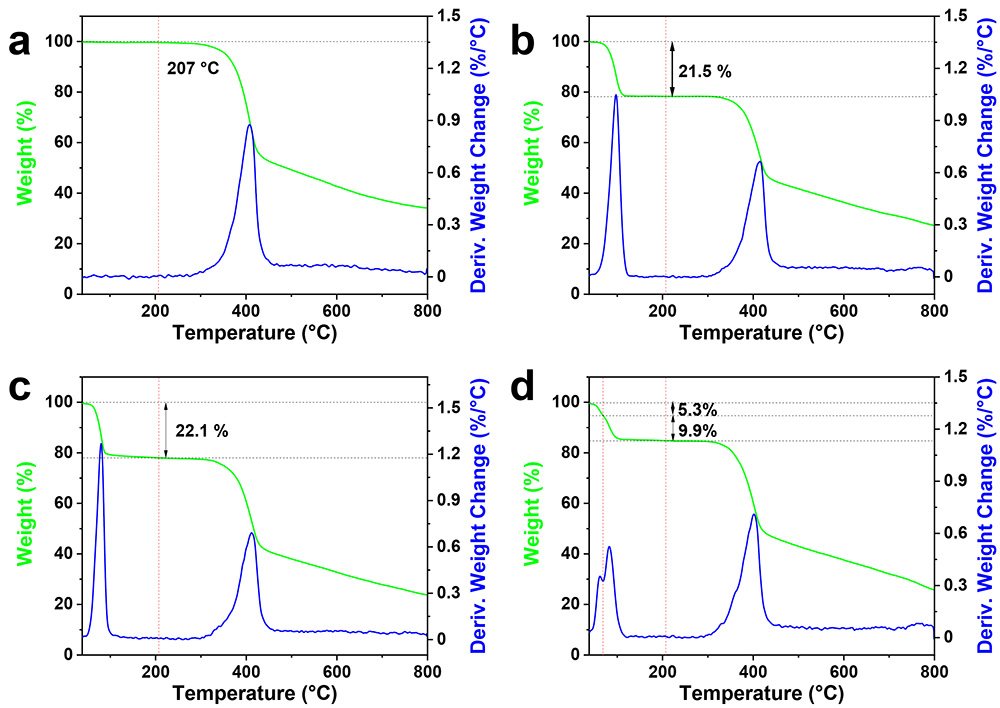


**Figure S4.** TGA of TPE-4OH crystals. (a) TPE-4OH powder. (b) The TGA of TPE-4OH microrods, (c) TPE-4OH microplates, and (d) TPE-4OH mesocrystals.


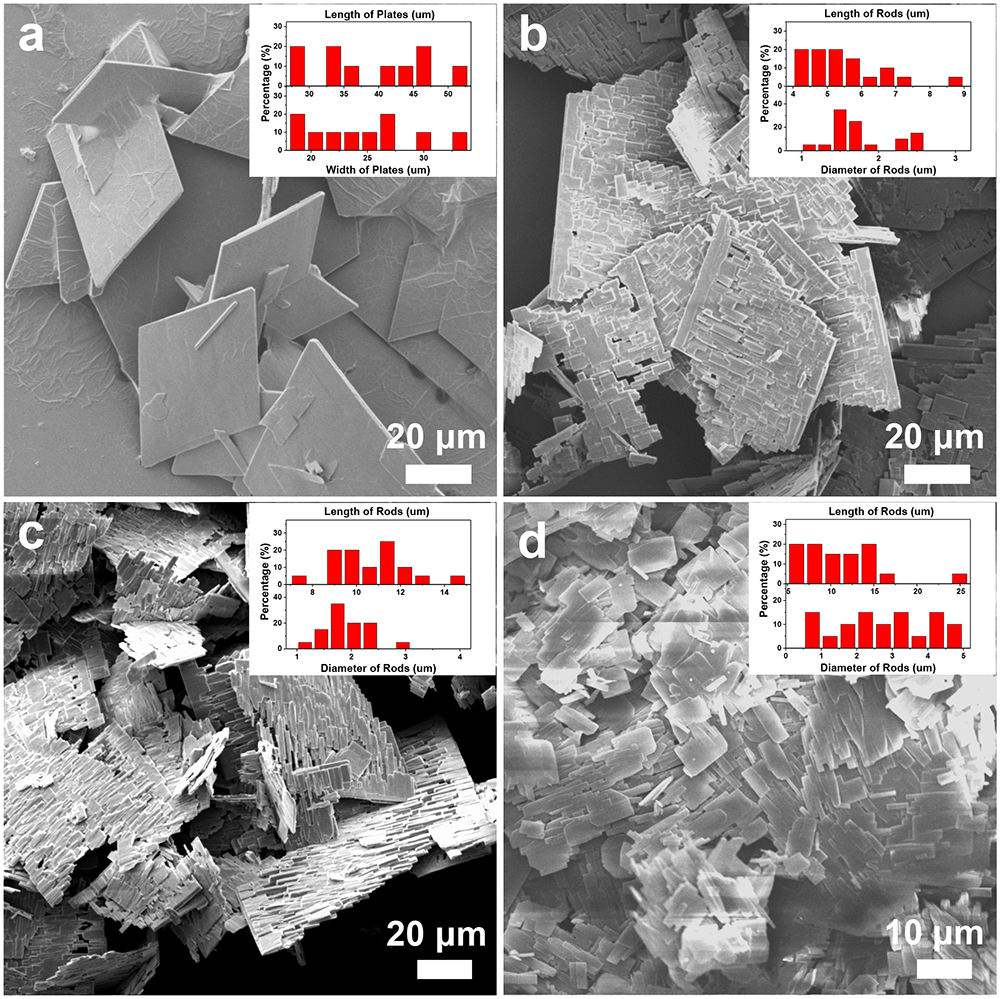


**Figure S5.** The SEM images of TPE-4OH crystals. (a-d) the SEM images of TPE-4OH microplates preserved in acetone/cyclohexane solution (V/V = 0.5/9.5, 1/9, 2/8, 3/7) for 8 mins, respectively. The inset images are the size distribution of TPE-4OH microplates preserved in acetone/cyclohexane solution (V/V = 0.5/9.5, 1/9, 2/8, 3/7) for 8 mins, respectively.

**
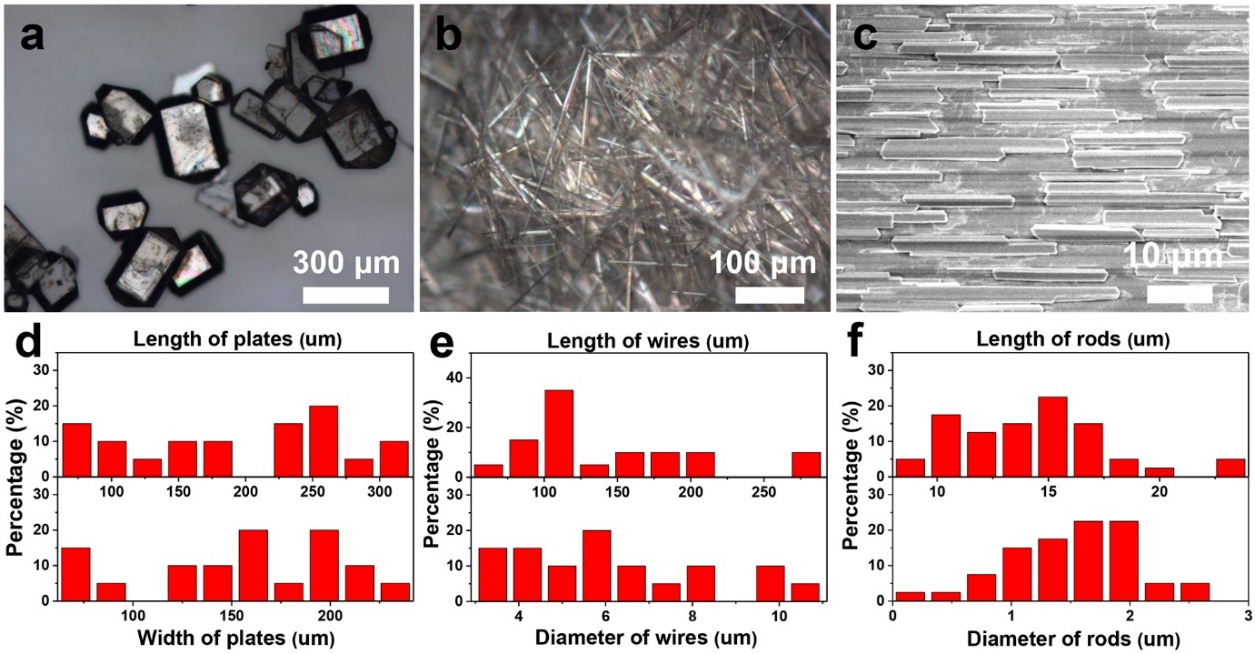
**

**Figure S6.** Optical images of (a) TPE-4Br microplates, (b) TPE-4Br microwires, and (c) TPE-4Br mesocrystals. (d-f) The size distribution of TPE-4Br microplates, TPE-4Br microwires, and TPE-4Br microrods on mesocrystals, respectively.


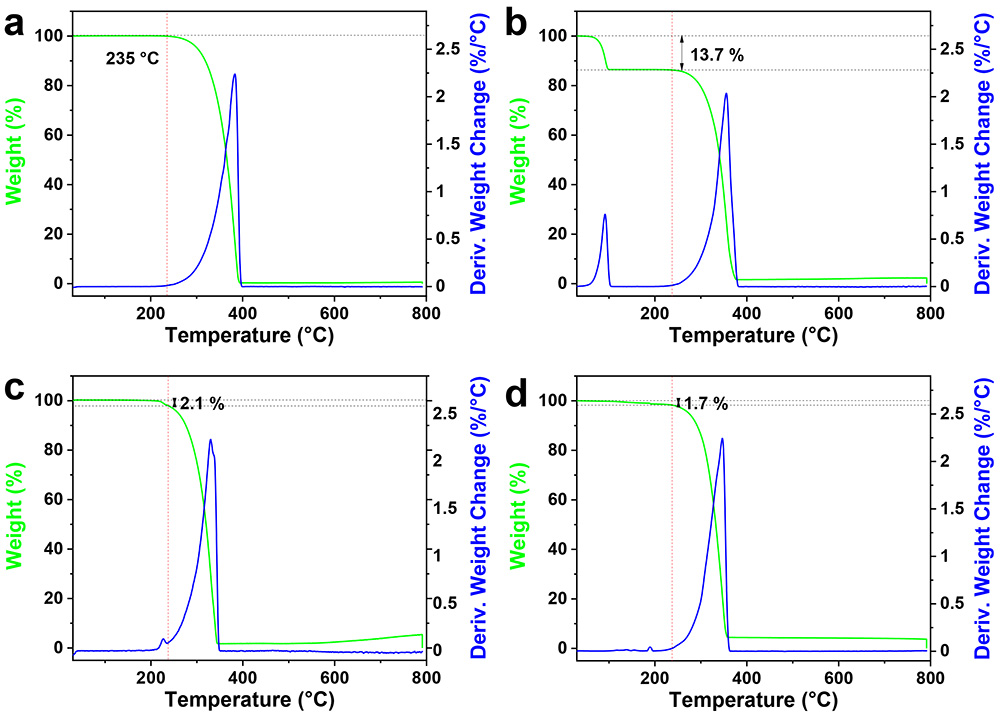


**Figure S7.** TGA of TPE-4Br crystals. TGA of (a) TPE-4Br powder. (b) TPE-4Br microplates, (c) TPE-4Br microwires, and (d) TPE-4Br microrod array mesocrystals.


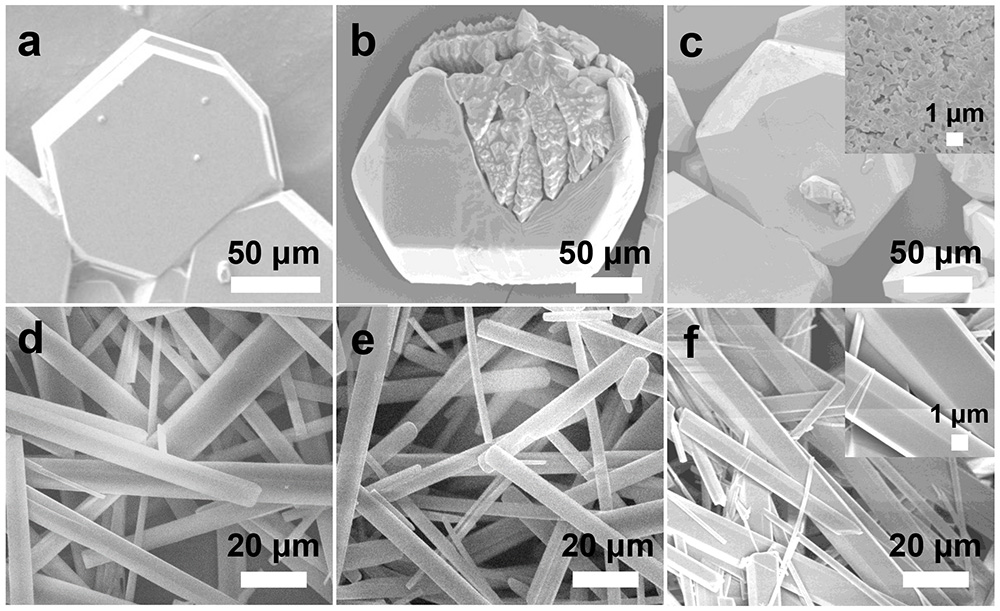


**Figure S8.** The stability of TPE-4Br crystals. SEM images of (a) TPE-4Br microplates, (b) the TPE-4Br microplates were purified 1 time by IPA. (c) The microplates were exposed in air for 2 h. (d) TPE-4Br microwires. (e) The microwires were purified 1 time by IPA. (f) The microwires were exposed in air for 2 h.


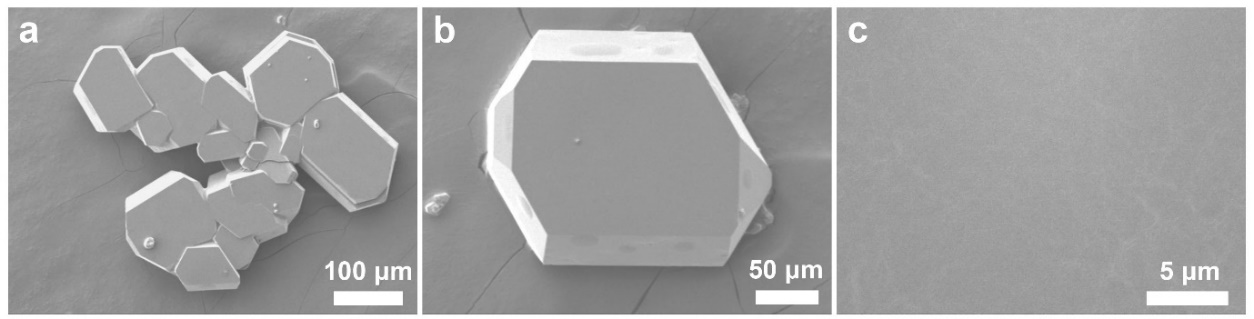


**Figure S9.** The stability of TPE-4Br crystals in preparative solutions for several weeks. SEM images of TPE-4Br microplates (a-c) with different amplification.


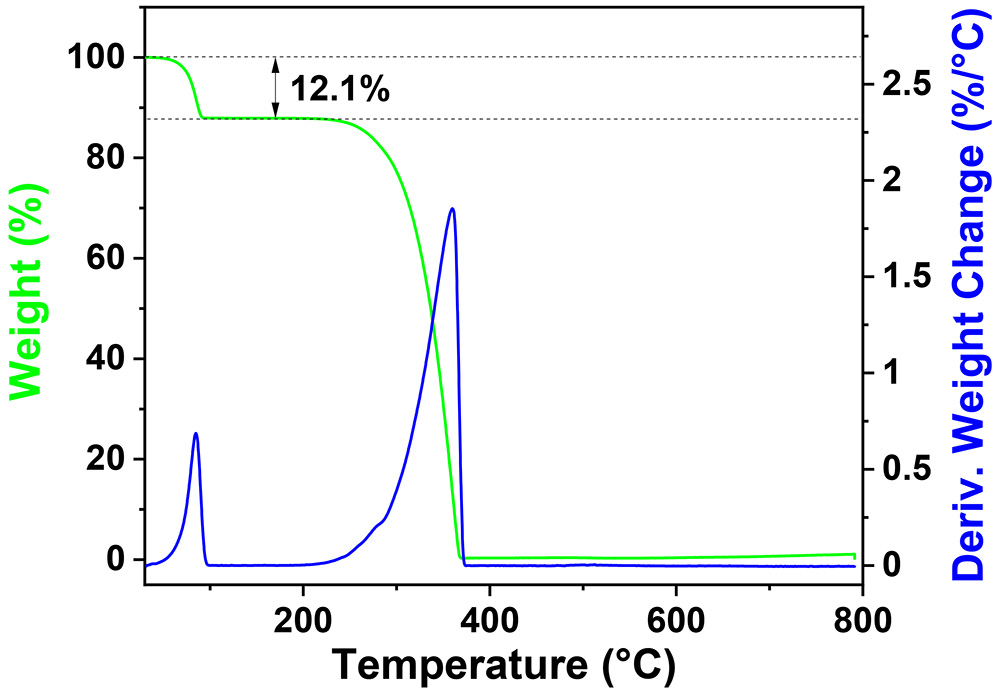


**Figure S10.** The TGA of TPE-4Br microplates after one round of purification by IPA solution.


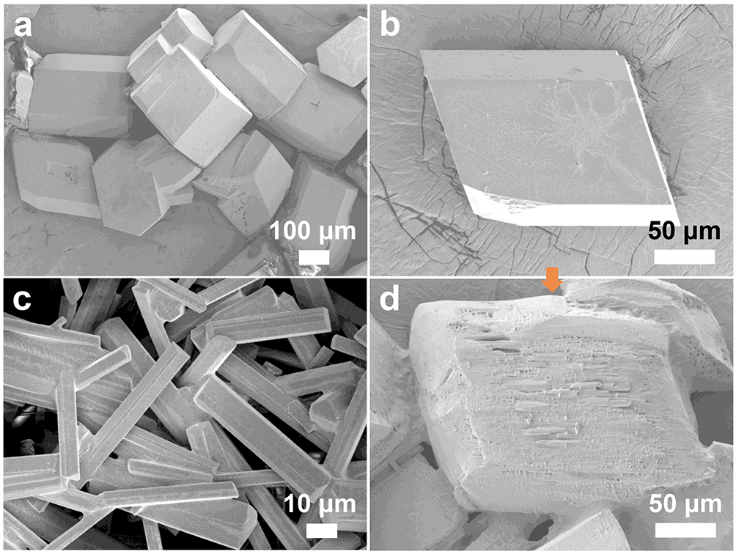


**Figure S11**. SEM images of (a, b) benzopinacol microplates. (c) benzopinacol microrods. (d) benzopinacol microrod arrays.

**Reference：**

Shrestha, L.K., Ji, Q., Mori, T., Miyazawa, K.I., Yamauchi, Y., Hill, J.P., and Ariga, K. (2013). Fullerene Nanoarchitectonics: From Zero to Higher Dimensions. *Chem. Asian J. 8*, 1662–1679.
